# The Chicago Veterinary College

**Published:** 1885-07

**Authors:** 


### THE CHICAGO VETERINARY COLLEGE.

On the 31st of March the closing exercises of this College were held. The attendance was quite liberal, a number of ladies lending their presence to the occasion. On the platform sat Profs. Baker, Withers, Sheppard, Periam and Hughes, while the graduates were ranged in front of the platform. Messrs. T. L. Armstrong, of Indianapolis; S. S. Baker, of Chicago; P. Quentman, of Chicago; Will J. Miles, of Charleston, Ill; J. S. Spangler, of Plainfield, Ill; A. Dean, of Girard, Mich., and A. S. Ziegler, of Lincoln, Ill.

The address to the graduating class, delivered by Prof. Withers, was full of feeling and sound advice as to the course to be pursued in establishing a practice. He complimented them on their energy, studious character and the tenacity with which they wrestled with the complex and complicated, till victory was assured. He spoke feelingly of the pleasant relations existing between the students and the faculty, and was sure that the class now going forth to battle for the cause of humanity and science would win honors and succes for themselves, and in so doing bestow honors upon their Alma Mater and help her to disseminate the seeds of learning broadcast. The Professor alluded to the vast extent of our territory, developed and to be developed, the vast field open for the educated veterinarian, the fact that the general public is rapidly becoming educated to the appreciation of the graduated veterinarian and a proper degree of deprecation of the would-be cure-all efforts of the "hoss" doctors and "rubbers." A number of useful hints and suggestions were given to the doctors, and the Professor then presented the diplomas.

The Valedictorian of the Class, Dr. S. S. Baker, then delivered his address. —*Horseshoer's and Hardware Journal.*

